# Editorial: Buffalo Health and Production

**DOI:** 10.3389/fvets.2021.810923

**Published:** 2022-02-11

**Authors:** Antonio Humberto Hamad Minervino, Domenico Vecchio, André Mendes Jorge, Selwyn Arlington Headley

**Affiliations:** ^1^Federal University of Western Pará, Santarém, Brazil; ^2^Istituto Zooprofilattico Sperimentale del Mezzogiorno Portici, Portici, Italy; ^3^São Paulo State University, São Paulo, Brazil; ^4^Department of Veterinary Preventive Medicine, Universidade Estadual de Londrina, Londrina, Brazil

**Keywords:** buffalo, animal health, animal reproduction, animal science, *Bubalus bubalis*, animal infectious diseases

The domestic buffalo (*Bubalus bubalis*), also known as water buffalo or Asian buffalo comprises two subspecies: the river buffalo (*B. bubalis bubalis*) and the swamp buffalo (*B. bubalis kerebau*) (1). Considering buffalo characteristics such as rusticity and productivity, we believe that buffalo production should be expanded worldwide, especially in developing countries with adequate natural conditions (2). This amazing and underused animal can become more productive and should be promoted as a target species to be used in smallholder and sustainable production systems (3).

To provide a venue for quality research on buffalo this Research Topic on ***Buffalo Health*** ***and Production*** has a deliberately broad scope to allow a wide range of articles to be published. A total of 29 papers are included, with three review articles, 21 original research articles, and five brief research reports from 223 authors from around the globe. Figure 1 presents a word cloud with the most common words used in the manuscript titles.

**Figure 1:**
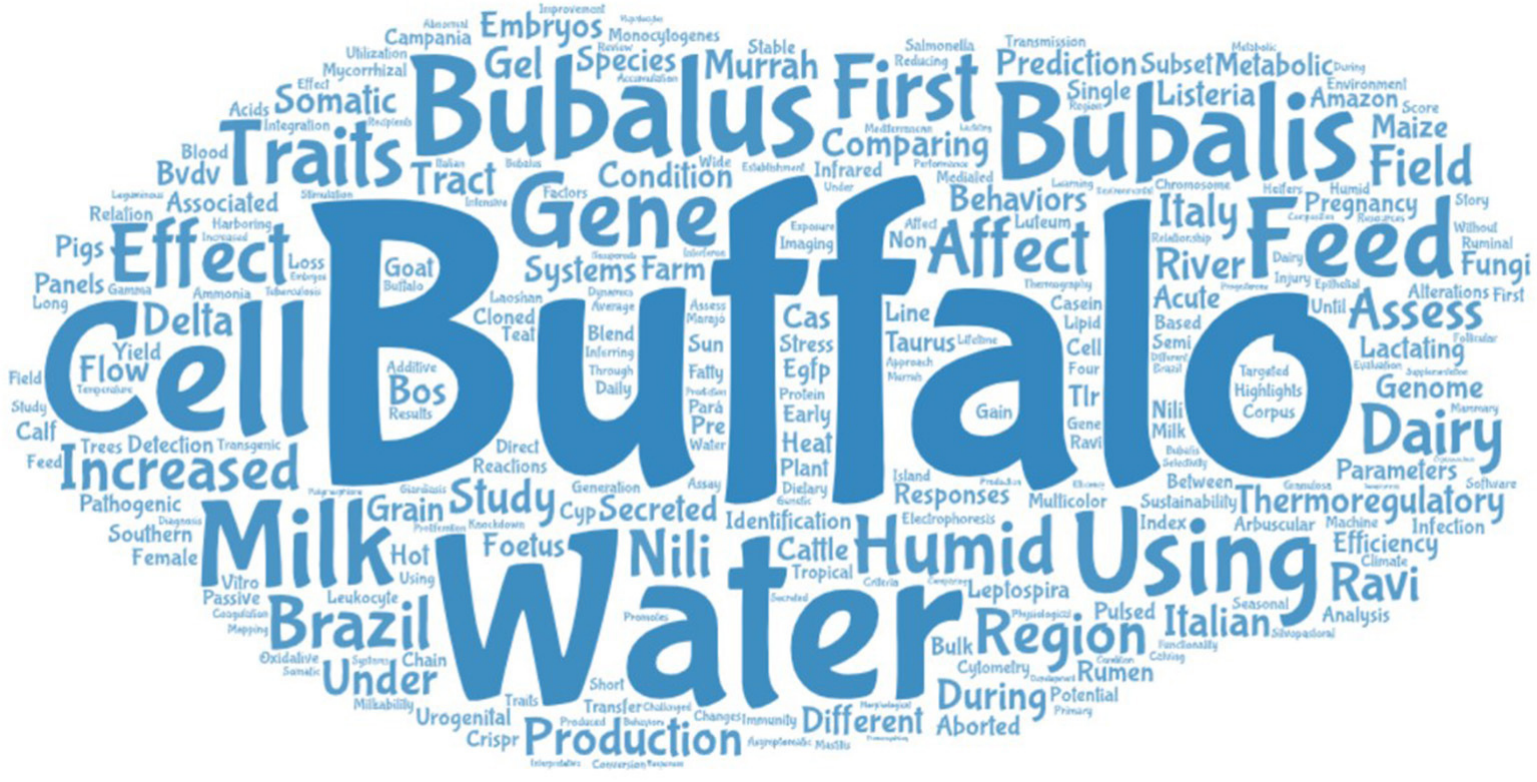
Word cloud created using the titles of the 29 articles published in the Research Topic: *Buffalo Health and Production*.

Two papers comprise comprehensive reviews of important buffalo diseases. The first review from de Barros et al. evaluates the available published literature of *Toxoplasma gondii* and *Neospora caninum* in buffalos and related serological evidence on these parasitic diseases in buffalos from most continents. The second review of parasite infections from de Aquino et al. presents an overview of the occurrence of cryptosporidiosis and giardiasis in water buffaloes and demonstrates that *Cryptosporidiu*m spp. and assemblages of *Giardia duodenalis* and may be sources for potential zoonotic infections. Moreover, the review of published data suggests that young buffalo are more prone to infection than their older counterparts and that infected buffalos can be asymptomatic or with clinical manifestations of these protozoan diseases.

An additional review paper from Minervino et al. presented the basic aspects of the water buffalo and unraveled the buffalo path followed from the origin of the species to its current global distribution, providing a more accurate estimate of the world buffalo count and distribution.

Considering original/brief research, the Research Topic published five articles related to infectious disease, one describing infection with *Leptospira* spp. in Brazil from Guedes et al. and an interesting study from Esposito et al. showing abortions due to *Listeria monocytogenes* in Italy, indicating that *Listeria* must be included as a possible cause of abortions in buffalo herds. The remaining infectious disease articles address new diagnostic tools to detect infections, such as the use of an interferon-gamma assay as a diagnostic strategy for *Mycobacterium bovis* in water buffalos from Martucciello et al.; a pulsed-field gel electrophoresis to detect Salmonella described by Santana et al.; and a flow cytometry to Study Leukocyte alterations during BVDV Acute Infection proposed by Grandoni et al..

The Research Topic included six physiopathology articles that address several aspects of buffalo, such as metabolic and lipid profiles (Zhang B. et al.), heat stress (Athaíde et al.), infrared thermography to assess thermoregulatory reactions (Brcko et al.), and passive immunity transfer (de Souza et al.). A highly viewed article from Sikka et al. employed machine learning algorithms for predicting feed conversion efficiency, using the blood parameters and average daily gain as predictor variables in buffalo heifers. The last study from Li et al. reported the seasonal dynamics of physiological, oxidative, and metabolic responses in buffaloes under hot and humid climates.

Four papers studied the nutritional and behavioral aspects of buffalo, focusing on different nutritional approaches and additives. One study from Galloso-Hernández et al. (a) evaluated the effect of silvopastoral systems in thermoregulation and feeding behaviors of buffalos indicating this system as an option for buffaloes under intense heat stress. A study from India (Chanu et al.) successfully developed a dietary supplementation of a plant-based feed additive that reduced ruminal ammonia and improved feed utilization efficiency and performance. Chiariotti et al. proposed that increasing the sustainability of maize grain does not affect the rumen of dairy buffalo. Finally, the selectivity of leguminous trees by water buffaloes in semi-intensive systems was evaluated by Galloso-Hernández et al. (b).

Four papers relate to dairy buffalo with a wide scope. An Italian article by Boselli et al. studied milkability and its relation to milk yield and somatic cell count in Mediterranean Italian Buffalo and proposed a classification to highlight some differences among curves that could have an impact on milk production and udder health. Costa, De Marchi, Visentin, et al. evaluated the effect of different types of pre-milking stimulation and an Italian article by Costa, De Marchi, Battisti, et al. evaluated the effect of the temperature-humidity index on buffalo milk. A Chinese article by Zhang H. et al. successfully established a new epithelial cell line that could be a useful tool for signal research and mammary gland bioreactors.

Three papers address the different genetic aspects of buffalo. A large study from Tamboli et al. evaluated the genetic parameters for the first lactation of Nili-Ravi Buffaloes and found higher heritability of first lactation traits, especially first peak milk yield, suggesting sufficient additive genetic variability. Other studies in the Research Topic evaluate the genetic aspects related to somatic cell score (Roldan-Montes et al.) and mastitis in water buffaloes (Jaiswal et al.).

Four articles presented important contributions to buffalo reproduction. A new technique proposed by Salzano et al. for early prediction of corpus luteum functionality was developed for the species using imaging software. The generation of transgenic cloned buffalo embryos was described by Zhao et al. using CRISPR/Cas9-mediated targeted integration. Saliba et al. focused on identifying which factors affect pregnancy until calving and pregnancy loss in buffalo recipients of *in vitro* produced embryos. One additional study by Lu et al. evaluated the effects of knockdown of the enzyme CYP19A1 in buffalo reproductive hormone secretion.

In conclusion, the Research Topic *Buffalo Health and Production* brings together a wide range of quality research focused on several aspects of buffalo health and production.

## Author Contributions

AM, DV, AJ, and SH were guest associated editors of the Research Topic and wrote and revised the editorial article. All authors contributed to the article and approved the submitted version.

## Conflict of Interest

The authors declare that the research was conducted in the absence of any commercial or financial relationships that could be construed as a potential conflict of interest.

## Publisher's Note

All claims expressed in this article are solely those of the authors and do not necessarily represent those of their affiliated organizations, or those of the publisher, the editors and the reviewers. Any product that may be evaluated in this article, or claim that may be made by its manufacturer, is not guaranteed or endorsed by the publisher.
